# Polyamidoamine Dendrimer Conjugates with Cyclodextrins as Novel Carriers for DNA, shRNA and siRNA

**DOI:** 10.3390/pharmaceutics4010130

**Published:** 2012-02-01

**Authors:** Hidetoshi Arima, Keiichi Motoyama, Taishi Higashi

**Affiliations:** Graduate School of Pharmaceutical Sciences, Kumamoto University, 5-1 Oe-honmachi, Kumamoto 862-0973, Japan; Email: motoyama@kumamoto-u.ac.jp (K.M.); higashit@kumamoto-u.ac.jp (T.H.)

**Keywords:** cyclodextrin, polyamidoamine dendrimer, conjugate, DNA delivery, shRNA delivery, siRNA delivery

## Abstract

Gene, short hairpin RNA (shRNA) and small interfering RNA (siRNA) delivery can be particularly used for the treatment of diseases by the entry of genetic materials mammalian cells either to express new proteins or to suppress the expression of proteins, respectively. Polyamidoamine (PAMAM) Starburst^TM^ dendrimers are used as non-viral vectors (carriers) for gene, shRNA and siRNA delivery. Recently, multifunctional PAMAM dendrimers can be used for the wide range of biomedical applications including intracellular delivery of genes and nucleic acid drugs. In this context, this review paper provides the recent findings on PAMAM dendrimer conjugates with cyclodextrins (CyDs) for gene, shRNA and siRNA delivery.

## 1. Introduction

Gene therapy has been utilized for vaccination and for the treatment of several diseases, such as genetic diseases, cancers, cardiovascular diseases, infectious diseases and dermatological diseases [1,2]. Approximately 500 clinical trials of gene therapy have been performed in the world from 2006 to 2010 [3]. Meanwhile, RNA interference (RNAi) technology has not only become a powerful tool for functional genomics, but also allows rapid drug target discovery and *in vitro* validation of these targets in cell culture. Selective gene silencing by RNAi can be achieved essentially by two nucleic acid based methods: (1) nuclear delivery of gene expression cassettes that express short hairpin RNA (shRNA); or (2) cytoplasmic delivery of small double-stranded interfering RNA oligonucleotides (siRNA) [4,5]. However, a standard therapeutic use of plasmid DNA (pDNA), shRNA and siRNA in clinical settings in humans has been hampered by the lack of effective methods to deliver these genes and nucleic acid drugs into the diseased organs and cells [6]. To address these issues, the improvement in transfer activity of a non-viral vector (carrier) is of utmost importance [7], although it is sure that viral vectors have become a major delivery system for shRNA [5]. 

Polycation-based genes and nucleic acid drug delivery methods have been strongly expected to offer sufficient efficiency in the transportation of therapeutic genes and nucleic acid drugs across various extracellular and intracellular barriers [8]. These barriers include the interactions with blood components, enzymatic degradation, excretion from kidney and sequestration by the reticuloendothelial system (RES) before reaching target cells. Cationic polymers constitute one of the most promising approaches to the use of non-viral vectors for gene and nucleic acid drug therapy. A better understanding of the mechanisms by which genes and nucleic acid drugs can escape from endosomes and, also, how genes enter the nucleus, has triggered new strategies of synthesis and has revitalized research into new polycation-based systems.

Polyamidoamine (PAMAM) Starburst^TM^ dendrimers (dendrimers), which are developed by Tomalia *et al.*, are biocompatible, non-immunogenic and water-soluble [9]. Dendrimers are a unique class of synthetic macromolecules having highly branched, three dimensional, nanoscale architectures with very low polydispersity and high functionality. These features have allowed their application in nanotechnology, pharmaceutical and medicinal chemistry to be attractive [10]. Since dendrimers possess terminal modifiable amine functional group, they form complexes with genes [11,12], shRNA [13,14,15] and siRNA [16,17,18] through the electrostatic interaction as well as the binding to glycosaminoglycans on cell surface [19]. As a result, dendrimers are known to offer efficient transfer activity for genes and nucleic acid drugs. In addition, the high transfection efficiency of dendrimers can be due to their well-defined shape and the proton-sponge effect. Herein, the proton-sponge effect is believed to be caused by cationic polymers that promote endosome osmotic swelling, disruption of the endosomal membrane and intracellular release of DNA and nucleic acid drugs [20]. Generally, it is evident that the nature of dendrimers as non-viral vectors depends significantly on their generations (G). Gene transfer activity of dendrimers with high generations is likely to be superior to that of low generations [21,22], although their cytotoxicity augment as their generations increase. Therefore, there has been a growing interest in developing dendrimers with low generations (<G4) because of their extremely low cytotoxicity [23]. 

Cyclodextrins (CyDs) were isolated approximately 100 years ago and were characterized as cyclic oligosaccharides [24,25,26]. The α-, β-, and γ-CyDs are the most common natural CyDs, consisting of six, seven, and eight glucose units, respectively. CyDs can improve the solubility, dissolution rate and bioavailability of drugs, and so the widespread use of CyDs is well known in the pharmaceutical field [27,28]. CyDs have been reported to interact with cell membrane constituents such as cholesterol and phospholipids, resulting in the induction of hemolysis of human and rabbit red blood cells (RRBC) [29,30,31], although CyDs cannot enter cells because of their high molecular weight (*ca*. 1,000) and hydrophilicity [26]. Regarding the delivery of genes and nucleic acid drugs using CyDs, it is acknowledged that CyDs interact with them only very slightly [32]. Thereby, the combination of CyDs with some cell-penetrating carriers was necessary to enter the cells. 

**Figure 1:**
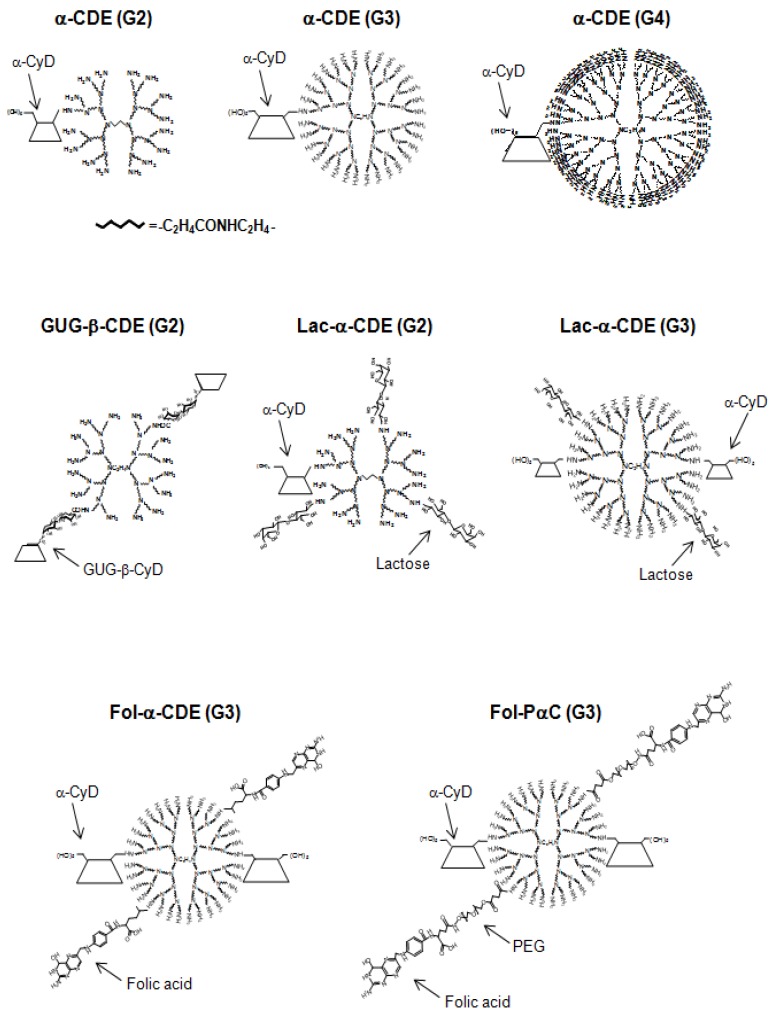
Chemical structures of various cyclodextrin/dendrimer conjugates (CDEs) used in this study.

Different strategies to promote interactions between CyD conjugates and genetic material have been exploited. Recently, Pack *et al.* [33] and Ortiz Mellet *et al.* [34] reported on CyD-based gene delivery systems. Intriguingly, Davis and co-workers have reported the potential uses of β-CyD-containing polycations (CDP) with adamantine-PEG or adamantine-PEG-transferrin for gene, DNAzyme and siRNA transfer [35,36,37,38]. It should be noted that the first targeted delivery of siRNA in humans via self-assembling, CyD polymer-based nanoparticles has been reported [39,40]. In addition, various CyD-appended polymers and polyrotaxanes have been acknowledged [41]; among these CyD-based polymers and supramolecules, such as pDNA, shRNA and siRNA delivery carriers. Arima and colleague originally developed various CyD conjugates with dendrimers (Figure 1).

## 2. α-CyD Conjugates with Dendrimer (α-CDE) as pDNA Carriers

It is necessary for non-viral carriers to possess high gene transfer activity, low cytotoxicity, negligible immunogenicity and adequate pharmacokinetic properties. To develop novel non-viral carriers to fulfill the criteria, we prepared dendrimers (G2, G3, G4) conjugates (CDE) with natural CyDs [42,43,44]. Herein, we employed the dendrimers having low generations because of their low cytotoxicity and the remaining proton-sponge effect [45]. Meanwhile, CyDs were used due to their prospective endosomal disrupting effects, through the release of membrane components from endosomal membranes after endocytosis, estimated from their hemolytic activity and liposomal membrane-disruptive effect [30,44]. To optimize the chemical structures of CDEs, we firstly prepared three CDE (G2) with α-, β- or γ-CyD at a molar ratio of 1:1 (dendrimer:CyD) [42]. Among them, dendrimers (G2) conjugates with α-CyD (α-CDE (G2)) elicited luciferase gene transfer activity approximately 100 times higher than dendrimers (G2) or non-covalent mixtures of dendrimer (G2) and α-CyD, when pDNA encoding luciferase gene was used [42]. Various α-CDEs, α-CDE (G3) with a degree of substitution (DS) of 2.4 (α-CDE (G3, DS 2)) was clarified to have best transfection efficiency with low cytotoxicity, *i.e.*, gene transfer activity of α-CDE (G3, DS 2) was found to be superior to that of TransFast™ (TF) and Lipofectin™ (LF), commercially-available transfection regents [43,44]. To clarify the enhanced gene transfer activity of α-CDE (G3, DS 2), Arima and colleague examined physicochemical properties, cellular uptake and intracellular distribution of pDNA complexes with α-CDE (G3, DS 2). The particle sizes and ζ-potential values of the pDNA complexes were equivalent to those of pDNA complexes with dendrimer (G3). The cellular uptake of the complexes with α-CDEs (G2, G3, G4) was comparable to that with dendrimer (G2, G3, G4). In addition, physical mixtures of the dendrimers (G3, G4)/pDNA complexes and α-CyD in the same stoichiometry of dendrimer and α-CyD (1:2.4 (molar ratio)) as that of α-CDE (G3, DS 2) did not change cellular uptake and gene expression of the pDNA complexes. These results suggest that some factors other than physicochemical properties or cellular uptake of pDNA complexes with α-CDE (G3, DS 2) may be potently associated with improving gene transfer activity. To reveal cellular uptake mechanisms of α-CDE (G3)/pDNA complexes, Arima and colleague examined the effects of various endocytosis inhibitors on cellular assciation of tetramethylrhodamine-5-(and 6)-isothiocyanate (TRITC)-α-CDE (G3) complex with fluorescein isothiocyanate (FITC)-pDNA and the colocalization of TRITC-α-CDE (G3), FITC-endocytosis markers, and FITC-pDNA after transfection using a flow cytometry and a confocal scanning laser microscopy in A549 cells, respectively. As a result, pDNA complexes with TRITC-α-CDE (G3, DS 2) colocalized with FITC-transferrin and FITC-cholera toxin B after transfection of pDNA complexes in the cells. Also, gene transfer efficiency of α-CDE (G3, DS 2) was inhibited by the addition of clathrin-dependent endocytosis inhibitors (chlorpromazine, sucrose) and raft-dependent endocytosis inhibitors (nystatin, filipin), but not a macropinocytosis inhibitor (amiloride) (unpublished data). These results suggest that α-CDE (G3, DS 2) is internalized via clathrin- and raft-dependent endocytosis. 

**Figure 2:**
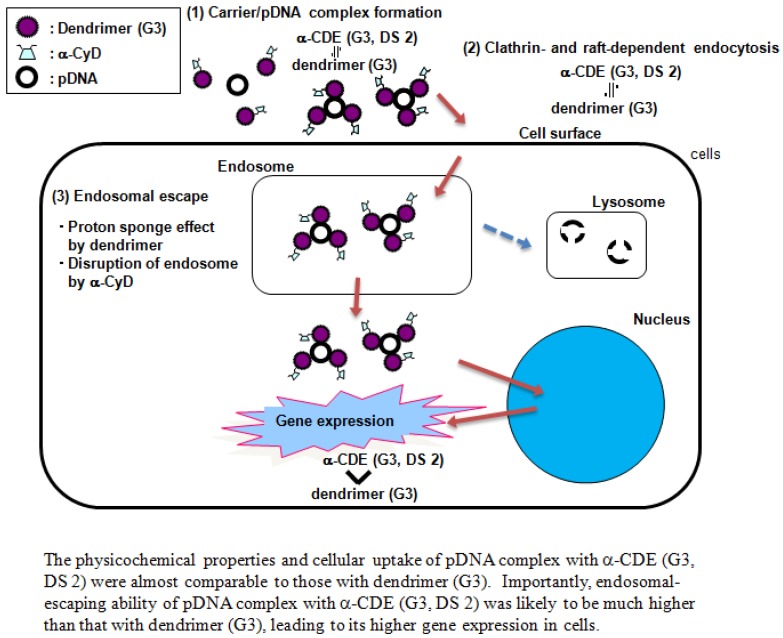
Proposed scheme for improved effects of gene transfer activity by α-CDE (G3, DS 2).

Next, we observed intracellular distribution of pDNA complex with α-CDE (G3, DS 2) using a confocal laser scanning microscopy. The fluorescence derived from FITC-pDNA in the α-CDE (G3, DS 2) system was observed in cytoplasm much more than that in the dendrimer system. Additionally, α-CDEs (G3, DS 2, 5) were found to disrupt liposomal membranes, model bilayer membranes, stronger than dendrimer (G3) and α-CDE (G3, DS 1). Collectively, these lines of evidence demonstrate that α-CDE (G3, DS 2) could be ascribed to the improved endosomal-escaping ability via the additive or synergetic effects of the proton-sponge effects of dendrimers and the endosomal membrane-disrupting effects of α-CyD as shown in Figure 2 [46]. However, transfection efficiency of the pDNA complexes with α-CDEs seems to be still low, probably due to the lack of the translocation ability of the carriers into nucleus [47]. Thus, translocation of the pDNA/α-CDE complex into nucleus should be improved in order to more increase gene expression.

Finding a safe and effective systemic delivery system is a major obstacle in gene therapy. Although viral vectors showed promise for high transfection rate, the immunogenicity associated with these systems has hindered further development. As an alternative to viral gene delivery, application of novel safe and effective polymeric systems that have shown high transgene expression when administered systemically has been expected [48]. Thereby, Kihara *et al.* evaluated gene transfer activity of α-CDE (G3, DS 2) after intravenous administration in mice. Twelve hours after intravenous administration of the solution containing pDNA complexes with α-CDE (G3, DS 2) at a dose of 50 μg pDNA/mice and at a charge ratio of 10 (carrier/pDNA), α-CDE (G3, DS 2) delivered pDNA more efficiently in spleen, liver, and kidney with negligible changes in blood chemistry data such as LDH, AST and BUN, compared with dendrimer and other α-CDE (G3, DS 1, 5). In particular, higher gene expression in spleen was observed 12 h after the administration of the pDNA complex with α-CDE (G3, DS 2). These results suggest the potential use of α-CDE (G3, DS 2) as a promising non-viral vector *in vitro* and *in vivo*, and these data may be useful for design of α-CyD conjugates with other non-viral vectors, although the further modification of the chemical structure of α-CDE (G3, DS 2) is required to improve the carrier’s ability.

## 3. GUG-β-CDE (G2) as DNA Carriers

As described above, gene transfer activity of α-CDE (G3, DS 2) should be improved *in vitro* and *in vivo*. As described below, Arima and colleagues recently reported that lactosylated α-CDE (Lac-α-CDE (G2)) and pegylated folate-appended-α-CDE (G3) (Fol-PαC (G3)) selectively deliver pDNA to hepatocytes and tumor cells *in vitro* and *in vivo*, respectively (Figure 1) [49,50]. These carriers have glucose and polyethylene glycol (PEG) as a spacer between dendrimer and targeting ligands, respectively, suggesting the importance of a spacer for a cell-specific pDNA delivery. However, it is still unknown whether introduction of a spacer between dendrimer and CyD improves gene transfer activity of α-CDEs. Therefore, Anno *et al.* used 6-*O*-α-(4-*O*-α-d-glucuronyl)-d-glucosyl-β-CyD (GUG-β-CyD) [51] as a novel branched CyD because of its high bioadaptability and low hemolytic activity in order to prepare various dendrimer (G2) conjugates with GUG-β-CyD (GUG-β-CDE (G2)) having different DS values of the glucuronyl-glucosyl group (Figure 1) [52].

**Figure 3:**
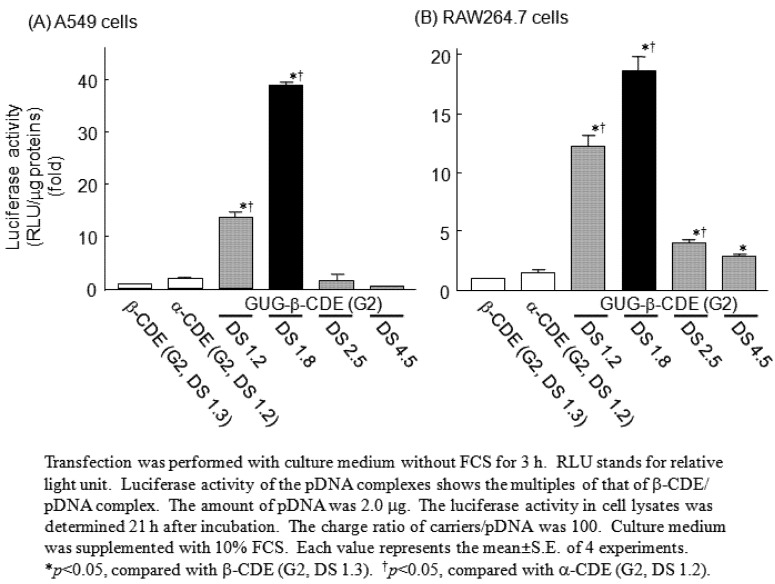
Transfection efficiencies of the pDNA complexes with α-CDE (G2, DS 1.2), β-CDE (G2, DS 1.3) and GUG-β-CDEs (G2, DS 1.2, 1.8, 2.5 or 4.5) in A549 cells and RAW264.7 cells.

Of the four GUG-β-CDEs (DS 1.2, 1.8, 2.5 and 4.5), GUG-β-CDE (G2, DS 1.8) showed the highest gene transfer activity *in vitro* (Figure 3). Actually, GUG-β-CDE (G2, DS 1.8) formed the complex with pDNA, although the complexation ability of GUG-β-CDE (G2, DS 1.8) may be slightly lower than that of α-CDE (G2, DS 1.2), β-CDE (G2, DS 1.3) or GUG-β-CDE (G2, DS 2.5, 4.5), possibly due to a decrease in the number of the positively charged primary amino groups of dendrimer or steric hindrance of GUG-β-CyD. The particle size of the pDNA complex with GUG-β-CDE (G2, DS 1.8) was about 190 nm, and the value was not significantly different from that of α-CDE (G2, DS 1.2) or β-CDE (G2, DS 1.3). In addition, the ζ-potential values of the pDNA complexes with carriers were in the range of 13 to 18 mV. The difference in ζ-potential values between GUG-β-CDE (G2, DS 1.8) and other carriers was rather slight. These results suggest that the particle size and the ζ-potential of the pDNA complex with GUG-β-CDE (G2, DS 1.8) are almost the same as those of α-CDE (G2, DS 1.2) and β-CDE (G2, DS 1.3), 1.3) systems to A549 and RAW264.7 cells. Taken together, these results suggest that some factors except for the physicochemical properties, enzymatic stability and cellular uptake of the pDNA complex with GUG-β-CDE (G2, DS 1.8) may be strongly involved in high gene transfer activity of GUG-β-CDE (G2, DS 1.8), compared to α-CDE (G2, DS 1.2) and β-CDE (G2, DS 1.3). 

Therefore, it may play an important role in gene transfer activity at the post-cellular uptake process of pDNA complex. Then, we observed the cells after transfection of pDNA complexes with TRITC-carriers for 6 h in A549 cells using a fluorescence microscope. In all systems, the fluorescence was observed over the whole cytoplasm, suggesting effective endosomal escape of the pDNA complexes after cellular uptake. However, the difference in the fluorescence intensity in cytoplasm was not visually obvious among these carriers. Interestingly, the fluorescence of TRITC-GUG-β-CDE (G2, DS 1.8) was strongly observed in the nucleus, compared with those of other carriers. The mechanism for nuclear localization of GUG-β-CDE (G2, DS 1.8) is still not clear. However, some lectins are known to exist in the nuclear membranes, and can recognize sugars such as glucose or galactose [53,54,55,56]. Additionally, the carbohydrate-recognition domain (CRD) of the lectin mainly recognizes 2-, 4-hydroxyl group of monosaccharides [47]. Hence, Anno *et al.* hypothecated that GUG-β-CDE (G2) having a 2-, 4-hydroxyl group in the spacer domain might be recognized by the nuclear lectins, and studied to address the hypothesis. As a result, Anno *et al.* revealed that the pDNA complex with GUG-β-CDE (G2, DS 1.8) shows high endosomal escaping ability and nuclear localization in A549 cells.

With few exceptions, where local administration is feasible, a progress towards broad clinical application of gene therapies requires the development of effective delivery systems. However, development of a novel non-viral vector suitable for systemic application is strongly expected [57]. Therefore, Anno *et al.* examined gene transfer activity 12 h after intravenous administration of the solution containing pDNA complex with GUG-β-CDE (G2, DS 1.8) to tail vein of mice [58]. Various carriers showed higher gene transfer activity in kidney than in other tissues. It is noteworthy that gene transfer activity of GUG-β-CDE (G2, DS 1.8) in kidney was much higher than that of α-CDE (G2, DS 1.2) or β-CDE (G2, DS 1.3) [58]. To investigate the safety profile of GUG-β-CDE (G2, DS 1.8), Anno *et al.* determined some blood chemistry data such as blood urea nitrogen (BUN) and aspartate aminotransferase (AST) 12 h after intravenous administration of its pDNA complexes in mice [58]. The parameters in the GUG-β-CDE (G2, DS 1.8) system were almost equivalent to those in the control system [58]. These results strongly suggest that the pDNA complex with GUG-β-CDE (G2, DS 1.8) has a safety profile even *in vivo*. Recently, gene therapy directly administered to blood vessels in patients with incurable renal diseases, such as Alport syndrome, polycystic kidney disease, renal cancers, glomerulonephritis and renal fibrosis has been studied. Potentially, the present findings suggest that GUG-β-CDE (G2, DS 1.8) might have the potential as a carrier for the gene therapy of kidney diseases, although it is necessary to reveal the detail gene expression region in kidney such as a glomerulus or a renal tubule. 

## 4. α-CDE (G3) as shRNA Carriers

Recently, shRNAs expression systems have been developed in order to prolong duration of the RNAi effect [59]. To elicit silencing in these systems, a small DNA insert encoding shRNA against the gene of interest is cloned into the vector downstream of the polymerase III promoter. Once transfected into mammalian cells, the insert-containing vector expresses the shRNA, which is rapidly processed by a Dicer-dependent cleavage in cytoplasm into siRNA, and then each is incorporated into the RNA induced silencing complex (RISC) followed by degradation of target mRNA [60]. As already described above, standard therapeutic use of RNAi in clinical settings in humans has, however, been hampered by the lack of effective methods to deliver the shRNA-expressing plasmid vectors into the diseased organs [61]. However, viral vectors have safety risks such as immunogenicity, oncogenicity and potential viral recombination that need to be solved [62]. For these reasons, the improvement in shRNA transfer activity of a non-viral vector (carrier) is of utmost important.

**Figure 4:**
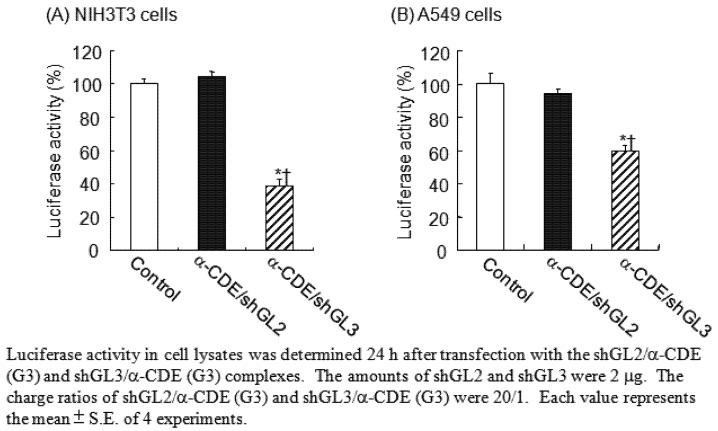
Sequence-specific gene-silencing effect of the complex of shGL2 or shGL3 with α-CDE (G3, DS 2) on luciferase activity in NIH3TE cells (**A**) and A549 (**B**) cells transiently expressing pGL3 luciferase gene.

The potential of α-CDE (G3, DS 2) as a novel carrier of pDNA expressing shRNA against pGL3 luciferase gene (shGL3) was evaluated. That is, the shGL3 transfer activity of α-CDE (G3, DS 2) was compared with that of dendrimer (G3). Regarding the complexation, α-CDE (G3, DS 2) formed a stable and condensed complex with shGL3 and induced a conformational transition of shGL3 from the B-form, a right-handed double helix with 10 bp per turn, to the C-form, a right-handed double helix with a 9.33 bp per turn that is less compact than the B-form of DNA, in solution, even in the low charge ratios. In addition, α-CDE (G3, DS 2) markedly inhibited the enzymatic degradation of shGL3 by DNase I. The shGL3 complex with α-CDE (G3, DS 2) at a charge ratio of 20/1 (carrier/shGL3) elicited the most potent RNAi effects in cells transiently and stably expressing the pGL3 luciferase gene without the off-target effects and cytotoxicity among the complexes with the various charge ratios (Figure 4). Besides, the RNAi effects were markedly enhanced by the further addition of the adequate amounts of siRNA to the shGL3 complex with α-CDE (G3, DS 2). Additionally, the prominent RNAi effects of the shGL3 complex with α-CDE (G3, DS 2) could be attributed to the stabilizing effect of α-CDE (G3, DS 2) on enzymatic degradation of shRNA and negligible cytotoxicity, although the formation of the stable complex may somewhat act as a negative factor. Collectively, these results suggest that α-CDE (G3, DS 2) possesses the potential to be a novel carrier for shRNA. 

## 5. α-CDE (G3) as siRNA Carriers

**Figure 5:**
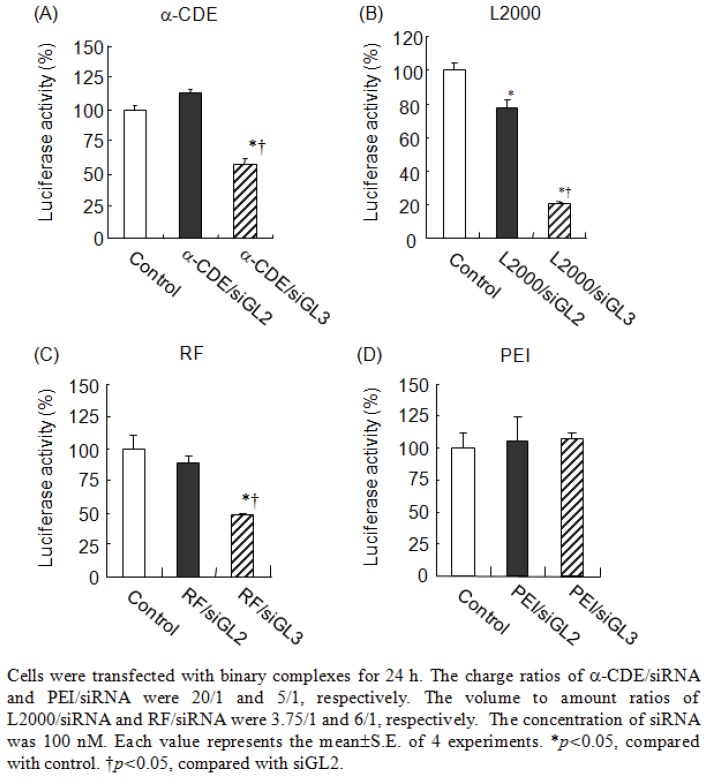
Inhibitory effects of binary complexes of carrirers/siRNA on luciferase activity in cells stably expressing luciferase gene (NIH3T3-luc cells).

**Figure 6:**
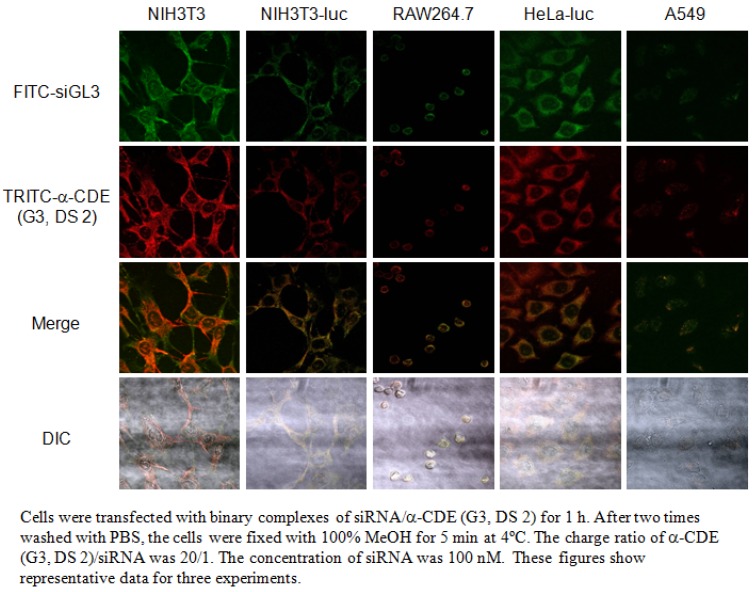
Instracelluar distribution of binary complexes of F1TC-siGL3/TRITC-α-CDE (G3, DS 2) into various cells.

As a first step, Tsutsumi *et al.* demonstrated the use of α-CDE (G3, DS 2) as siRNA carriers, since the siRNA complex has efficient RNAi effects with negligible cytotoxicity in the ternary complex system of pDNA/siRNA/α-CDE (G3, DS 2). Tsutsumi *et al.* demonstrated the complex exists in cytoplasm and the lack of translocation into nucleus [63]. As the second step, Tsutsumi *et al.* demonstrated the potential use of α-CDE (G3, DS 2) as a siRNA carrier in the binary system of siRNA/α-CDE (G3, DS 2), because the siRNA complex has efficient RNAi effects on firefly luciferase gene expression with negligible cytotoxicity, compared to the binary system of siRNA complexes with Lipofectamine^TM^2000 (L2000), RNAiFect^TM^ (RF) and linear polyethyleneimine (PEI) (Figure 5) [64]. Next, Arima *et al.* studied the RNAi effects of the siRNA/α-CDE (G3, DS 2) complexes on endogenous gene expression, as well as physicochemical properties, cytotoxicity, local irritation, interferon response, cellular uptake and intracellular distribution of the siRNA complexes. As a result, the siRNA complex with α-CDE (G3, DS 2) showed potent RNAi effects against Lamin A/C and Fas expression with negligible cytotoxicity, compared to those of the transfection reagents in Colon-26-luc cells and NIH3T3-luc cells, which stably express pGL3 luciferase gene [65]. Interestingly, α-CDE (G3, DS 2) delivered fluorescent-labeled siRNA to cytoplasm, not nucleus, after transfection in NIH3T3-luc cells (Figure 6), consistent with the pDNA complex with α-CDE (G3, DS 2). Furthermore, α-CDE (G3, DS 2) suppressed siRNA degradation by serum. Strikingly, the α-CDE (G3, DS 2)/siRNA complex exerted the *in vivo* RNAi effect on pGL3 luciferase expression in Colon-26-luc cells after not only intratumoral, but also intravenous administrations in tumor-bearing mice. Additionally, the blood chemistry values did not change after intravenous injection of α-CDE (G3, DS 2)/siRNA complex at the same siRNA dose as that showing the RNAi effect. This *in vivo* safe profile of the α-CDE (G3, DS 2)/siRNA complex is highly likely to be consistent with the *in vitro* safe profile, e.g. negligible cytotoxicity and hemolytic activity. These results suggest the potential use of α-CDE (G3, DS 2) as a novel carrier for siRNA both *in vitro* and *in vivo*. 

## 6. Potential Use of Polypseudorotaxanes (PPRXs) of Pegylated Dendrimers (PEG-Dendrimers) with CyDs as the Novel Sustained Release Systems for pDNA

CyD-based polyrotaxanes and PPRXs have inspired interesting exploitation as novel biomaterials because of their low cytotoxicity, controllable size, and unique architecture. Actually, the potential applications of CyD-based polyrotaxanes and PPRXs in life science and biotechnology have been reported [66].

Motoyama *et al.* demonstrated the potential use of PPRXs of PEG (molecular weight 2000)-grafted dendrimer (PEG-dendrimer) with CyDs as novel sustained release systems for pDNA [67]. The PEG-dendrimer/pDNA complex formed PPRXs with α-CyD and γ-CyD, but not with β-CyD, in solutions. In the PEG-dendrimer/CyDs PPRXs systems, 17.9 mol of α-CyD and 8.8 mol of γ-CyD were determined to be involved in the PPRXs formation with one PEG chain by α-CyD and γ-CyD, respectively, in the ^1^H-NMR study. In addition, the CyDs PPRX formation resulted in the sustained release of pDNA from PEG-dendrimer complex with pDNA at least 72 h *in vitro*. In addition, the release of pDNA from CyDs PPRX retarded as the dissolution medium volume decreased. These results suggest that the PEG-dendrimer/CyD PPRX systems can work as a sustained pDNA release system. Potentially, the PPRX formation with CyDs may be useful as a sustained drug delivery technique for other pegylated polymers [67]. Most recently, Motoyama *et al.* clarified that pegylated α-CDE/CyD PPRXs are promising sustained release systems *in vitro* and *in vivo* [68].

## 7. Functionalized α-CDEs (G2, G3) for Cell-Specific DNA Carriers

**Figure 7:**
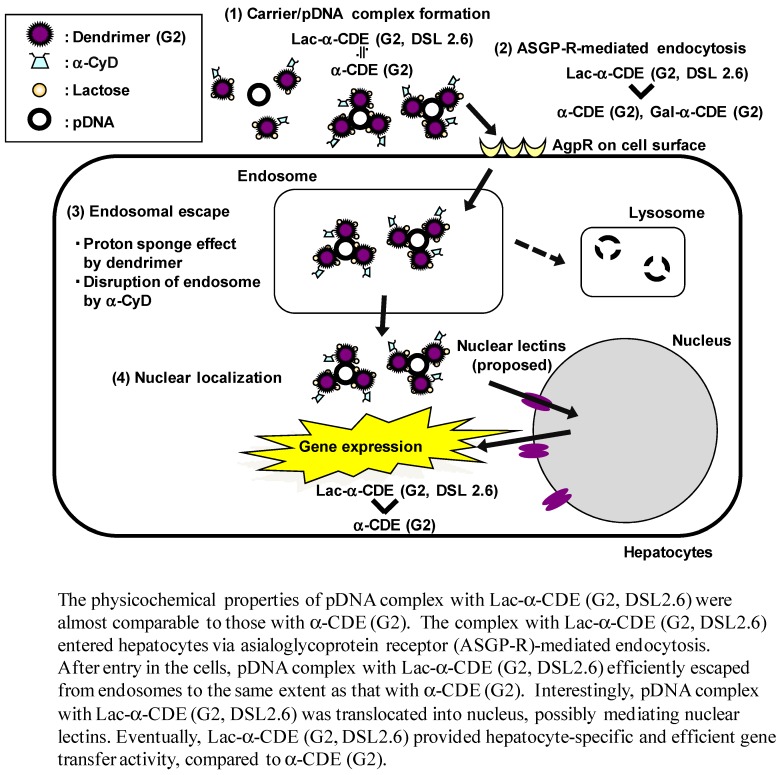
Proposed scheme for improved effects of gene transfer activity by Lac-α-CDE (G2, DSL 2.6).

**Figure 8:**
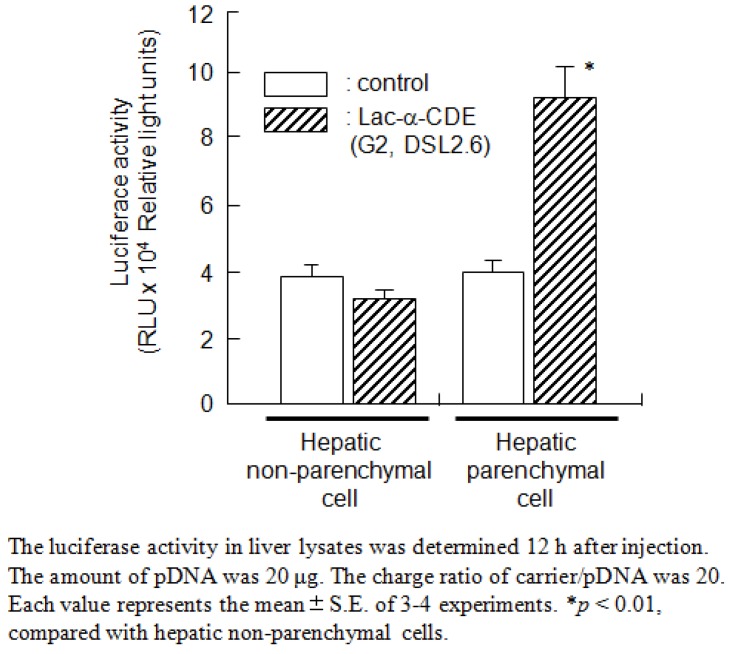
Luciferase activity in hepatic non-parenchymal cells or hepatic parenchymal cells 12 h after intravenous administration of the pDNA complexes with Lac-α-CDE (G2, DSL 2.6).

As described above, we prepared the three types of sugar-appended α-CDEs: mannosylated α-CDEs (Man-α-CDEs (G2, G3)) [47,69], galactosylated α-CDEs (Gal-α-CDEs (G2)) [70] and lactosylated α-CDEs (Lac-α-CDE (G2, G3)) [49,71] with various degrees of substitution (DS) of these sugar moieties. The DS values of mannose and lactose moieties are abbreviated to DSM and DSL, respectively. Man-α-CDE (G3, DSM 10) has less cytotoxicity and prominent gene transfer activity through its serum resistant ability, endosome-escaping abilities, and nuclear localization ability. Unexpectedly, Man-α-CDE (G3, DSM 10) did not, however, elicit cell-specific gene delivery. Likewise, Gal-α-CDEs (G2) provided similar advantageous properties as Man-α-CDE (G2), but lacked cell-specificity. The reason why the Man-α-CDEs (G2, G3) and Gal-α-CDEs (G2) lacked recognition to cell-surface lectins is still unclear, but the spacer was possibly the cause, because Man-α-CDE (G3, DSM 10) and Gal-α-CDEs (G2) have the same spacer, phenyl isothiocyanate. Arima and colleagues are currently preparing sugar-appended α-CDEs with the new spacer in order to specifically and strongly bind cell surface lections. Meanwhile, Arima *et al.* recently reported that Lac-α-CDE (G2, DSL 2.6) was found to have much higher gene transfer activity than dendrimer (G2), α-CDE (G2), Lac-α-CDE (G2, DSL 1.2, 4.6, 6.2, 10.2) and lactosylated dendrimer (Lac-dendrimer (G2), DSL 2.4) in HepG2 cells. Importantly, cell-specific gene transfer activity of Lac-α-CDE (G2, DSL 2.6) was dependent on the expression of cell-surface asialoglycoprotein receptor (ASGP-R), although the physicochemical properties of pDNA complex with Lac-α-CDE (G2, DSL 2.6) were almost comparable to those with α-CDE (G2). Additionally, we revealed that the free fraction of Lac-α-CDE (G2, DSL 2.6), which was not involved in the complex with pDNA, enhanced gene transfer activity of Lac-α-CDE (G2, DSL 2.6). Moreover, Arima *et al.* observed nuclear localization of pDNA complex with Lac-α-CDE (G2, DSL 2.6) after transfection in HepG2 cells. Taken together, these results suggest that hepatocyte-specific gene transfer activity of Lac-α-CDE (G2, DSL 2.6) is attributed to ASGP-R-dependency, efficient endosomal-escaping ability, nuclear localization and negligible cytotoxicity (Figure 7). It should be noted that Lac-α-CDE (G2, DSL 2.6) provided much higher gene transfer activity in hepatic parenchymal cells than in hepatic non-parenchymal cells 12 h after intravenous administration in mice (Figure 8). In addition, gene transfer activity of Lac-α-CDE (G2, DSL 2.6) to hepatocytes was significantly higher than that of JetPEI^TM^-Hepatocyte with negligible changes in blood chemistry values 12 h after intravenous administration in mice. These results hold promise for the potential use of Lac-α-CDE (G2, DSL 2.6) as a hepatocyte-selective non-viral vector with negligible cytotoxicity. Arima and colleagues are now studying whether Lac-α-CDE (G3) is useful for a hepatocyte-selective siRNA carrier to treat certain genetic diseases.

**Figure 9:**
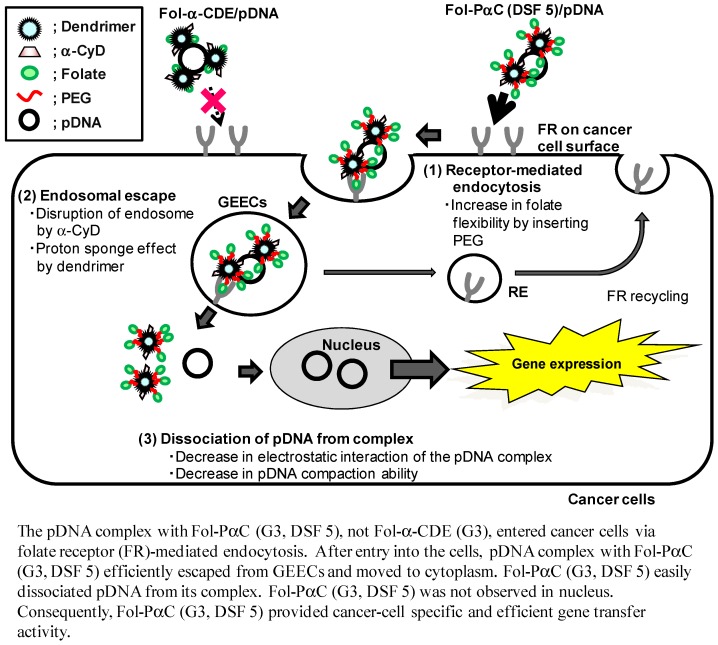
Proposed scheme for cancer cells-selective gene transfer activity of Fol-PαC (G3, DSF 5)/pDNA complex.

Folic acid (FA) has been shown to be one of the most promising ligands for targeting a range of human carcinomas, including breast, ovary, endometrium, kidney, lung, head and neck, brain and myeloid cancers, which are known to express folate receptors (FR). In an attempt to develop FR-overexpressing cancer cell-specific gene transfer carriers, we recently prepared folate-appended α-CDEs (Fol-α-CDE (G3)) and folate-PEG-appended α-CDEs (Fol-PαC (G3)) and evaluated their potential as novel cancer cell-specific gene transfer carriers (Figure 1) [50]. Eventually, we revealed that Fol-PαC (G3, DS of pegylated folate (DSF) 5) could be used as a FR-overexpressing cancer cell-selective gene transfer carrier, compared to Fol-α-CDE (G3), because of FR-mediated gene delivery and the extremely low cytotoxicity (Figure 9). There is a recent review paper describing the potential uses of Lac-α-CDEs (G2, G3) and Fol-PαC (G3) for siRNA carriers [72].

## 8. Conclusion

Many attempts have been made to design and evaluate CyD conjugates with polymers for DNA, shRNA and siRNA carriers. In this review, the potential of α-CDEs as DNA, shRNA and siRNA carriers were demonstrated for the first time. Secondly, GUG-β-CDE (G2, DS 1.8) was described to be a preferable pDNA carrier to α-CDE (G2, DS 1.2). Thirdly, the PEG-dendrimer or PEG-α-CDE/CyD PPRX systems were shown to work as a sustained pDNA release system. Fourthly, the potential use of lactosylated and pegylated folate-appended α-CDEs as targeting carriers to hepatocytes- and cancer cells, respectively, was introduced. Thus, these α-CDEs and GUG-β-CDEs are likely to be promising carriers for pDNA, siRNA and shRNA, but their potency may be insufficient for clinical use. Further improvement of the potency of α-CDE (G3, DS 2) and GUG-β-CDE (G2, DS 1.8) as carriers for DNA, shRNA and siRNA should be performed. Elaborate studies are further required to develop novel carriers for genes and various nucleic acid drugs such as shRNA, siRNA, decoy DNA, antisense DNA, ribozyme and aptamers. Most recently, we revealed that fucosylated α-CDE shows good potential as a decoy DNA carrier. The future should see various clinical use products using CyD-containing carriers for DNA, shRNA and siRNA.
